# Beetles that live with ants (Carabidae, Pseudomorphini, *Pseudomorpha* Kirby, 1825): A revision of the *santarita* species group

**DOI:** 10.3897/zookeys.362.6300

**Published:** 2013-12-13

**Authors:** Terry L. Erwin, Lauren M. Amundson

**Affiliations:** 1 Hyper-diversity Group, Department of Entomology, MRC-187, National Museum of Natural History, Smithsonian Institution, Washington, P.O. Box 37012, DC 20013-7012, USA; 2 Hyper-diversity Group Summer Intern, Department of Entomology, MRC-187, National Museum of Natural History, Smithsonian Institution, Washington, P.O. Box 37012, DC 20013-7012, USA

**Keywords:** False-form beetles, new species, new species groups, identification key, distributions, male genitalia, female ovipositor, Hymenoptera: Formicidae

## Abstract

The Western Hemisphere genus *Pseudomorpha* Kirby 1825 was last revised by Notman in 1925 based on only a few known species (22) and paltry few specimens (73); other authors have added an additional six species represented by 53 additional specimens since 1925. Baehr (1997) assigned three species from Australia to this genus, albeit in a new subgenus, *Austropseudomorpha* Baehr 1997. A recent study of collections from throughout the Americas (1757 specimens) has revealed numerous new species that can be arrayed across 19 species groups based on a suite of attributes, some used by Notman and others newly discovered. A taxonomic revision of the species contained in one of these species groups, *santarita*, is provided herein, as well as a distributional synopsis of the remaining 18 species groups. New species described herein are as follows, each with its type locality: *Pseudomorpha huachineras* **p. n.**, Arroyo El Cocono, Sierra Huachinera, Sonora, México; *P. patagonia* **sp. n.**, Madera Canyon, Santa Rita Mountains, Arizona; *P.penablanca* **sp. n.**, Peña Blanca Lake, Arizona; *P. pima* **sp. n.**, Madera Canyon (lower), Santa Rita Mountains, Arizona; *P. santacruz* **sp. n.**, Madera Canyon, Santa Rita Mountains, Arizona; and *P. santarita* **sp. n.**, Santa Rita Ranch, Santa Rita Mountains, Arizona.

## Abstract (es)

El género *Pseudomorpha* Kirby 1825 del hemisferio occidental fue revisado la última vez por Notman en 1925 basado en solo unas pocas especies conocidas (22) y pocos especímenes muy pobres (73); desde 1925 otros autores añadieron 6 especies representadas por 53 especímenes adicionales. Baehr, en 1997, asignó tres especies de Australia a este género, aunque en un subgénero nuevo, *Austropseudomorpha* Baehr 1997. Un estudio reciente de las colecciones a través de las Américas (1757 especímenes) ha revelado numerosas especies nuevas que pueden ser ordenadas en 19 grupos de especies basadas en una serie de atributos, algunos usados por Notman y otros recién descubiertos. Una revisión taxonómica de las especies contenidas en uno de estos grupos de especies, *santarita*, se provee aquí, así como una sinopsis distribucional de los otros 18 grupos. Las nuevas especies descritas aquí son las siguientes, cada una con su localidad tipo: *Pseudomorpha huachinera* **sp. n.**, Arroyo El Cocono, Sierra Huachinera, Sonora, México; *P. patagonia* **sp. n.**, Madera Canyon, Santa Rita Mountains, Arizona; *P. penablanca* **sp. n.**, Peña Blanca Lake, Arizona; *P. pima* **sp. n.**, Madera Canyon (bajo), Santa Rita Mountains, Arizona; *P. santacruz*, **sp. n.**, Madera Canyon, Santa Rita Mountains, Arizona; and *P. santarita* **sp. n.**, Santa Rita Ranch, Santa Rita Mountains, Arizona.

## Introduction

Species of *Pseudomorpha* and some related genera are obligatory myrmecophiles in their larval stages. All known species of *Pseudomorpha* are terrestrial and their ant hosts live in the soil; however, adults of the pseudomorphine genus *Samiriamorpha* have been found in the arboreal nests of *Azteca* ants (Erwin and Geraci 2008). The genus *Pseudomorpha* currently includes 27 described species in the Western Hemisphere and more than 125 undescribed species (Erwin in prep.). The present contribution provides information on six undescribed species in the new *santarita* species groups with a key for identification of those species. Notman (1925) did not have specimens of this group; therefore, they do not run to any couplet he provided in his key. With the current treatment, we start bringing organization to one of the last poorly known carabid lineages in North America particularly, but also in Middle and South America. This information will begin to aid those interested in ants and their commensals, as well as collection managers in understanding what is present in their collections and how it is to be ordered.

Erwin and Geraci (2008) provided a complete history of work on this group of carabid beetles in the Western Hemisphere; Baehr (1992, 1997) did the same for the Eastern Hemisphere species. The only monograph published of the Western Hemisphere species is that of Notman (1925). His key to the species known to him at the time, as it turns out, is more of a key to species groups now that we know there are over 125 species in *Pseudomorpha*, alone. Lenko (1972) and Erwin (1981) described larvae and Liebherr and Kavanaugh (1985) noted that species of *Pseudomorpha* are ovoviviparous. Since Erwin and Geraci (2008), an additional new genus was discovered in Guyane (French Guiana) (Erwin 2013)

## Specimens and methods

Included in the overall study of this genus are a total of 1757 specimens from the National Museum of Natural History, Washington, DC (NMNH, Terry L. Erwin, Curator) and several other institutions and private collections (see Appendix 1 for the specimens covered in the current paper).

Methods and species concepts follow those previously described (Ball 1959; Erwin and Kavanaugh 1981; Kavanaugh and Erwin 1991). The species validation and diagnosis format follows as closely as possible that suggested in Erwin and Johnson (2000). Measurements of length (ABL, SBL) and width (TW) follow those of Ball (1972) and Kavanaugh (1979): ABL (apparent body length), measured from apex of labrum to apex of the abdomen; SBL (standardized body length), equals the sum of the lengths of the head (measured from apex of clypeus to a point on midline at level of the posterior edge of compound eyes), PL (pronotal length ), measured from apical to basal margin along midline, and LE (elytron length), measured from apex of scutellum to apex of the longer elytron; and TW (total width), measured across both elytra at their widest point with suture closed. Measures and ratios are presented in the tables in Appendix 2.

Habitus and attribute images of the adult beetles portray most of the character states referred to in the key provided. Male and female genitalic presentations are standard for descriptive taxonomy of carabid beetles, and in this case are digital photo-illustrations (Erwin 2011). The images of the adult and its parts were made with a Visionary Digital^TM^ high resolution imaging system. Figure captions include an ADP number, which is a unique identification number for the specimen that was illustrated or imaged and links the specimen and associated illustrations and/or image to additional information in electronic databases at the NMNH.

Geographical data are presented based on all known specimens of each species available at the time of manuscript preparation. Georeferences have been determined from locality information provided on specimen labels. Latitude and longitude are reported in decimal degrees. A distribution map is provided for the species (Fig. 18). Herein, an English vernacular name is proposed, as vernacular names are becoming increasingly needed in conservation and/or agricultural and forestry applications, as well as for the Encyclopedia of Life (www.eol.org).

## Accounts of taxa

### Pseudomorphini

Newman, 1842

http://species-id.net/wiki/Pseudomorphini

False-form beetles

Pseudomorphini Newman, 1842:365 (as Pseudomorphites)

#### Taxonomy.

Stable at the generic level.

#### Classification.

According to Ober and Maddison (2008), Pseudomorphini appears as a branch of the higher Carabidae and associated with Graphipterini and Orthogonini; according to Erwin and Geraci (2008), the adelphotaxon is the tribe Orthogonini. All three tribes are associated in some way with ants or termites. Male genitalia of pseudomorphines have a bonnet-shaped phallobase like the lebiomorphs, yet their accompanying parameres are large and nearly symmetrical (and in some species the parameres are sparsely setiferous, as in some primitive lineages of the family). Many known lineages of Pseudomorphini have been so highly selected for life with ants (and possibly termites) that external structures do not help much in discovering more normal carabid relatives.

#### References.

Baehr (1992, 1997); Erwin and Geraci (2008); Moore (1964, 1974, 1983); Ogueta (1967); Notman (1925).

### Pseudomorpha

Kirby, 1825

http://species-id.net/wiki/Pseudomorpha

Pseudomorpha Kirby, 1825:98
Heteromorpha Kirby, 1825:109
Axinophorus Dejean, 1829:174
Drepanus Dejean, 1831:434
Heteromorphus Chaudoir, 1852:63

#### Type species.

*Pseudomorpha excrucians* Kirby, 1825:101

#### Proposed English vernacular name.

Western False-form beetles

#### Number of described Western Hemisphere species.

27

#### Current known number of undescribed Western Hemisphere species.

125

#### Number of described Australian species.

3 (Subgenus *Austropseudomorpha* Baehr, 1997)

#### Adelphotaxon.

*Tuxtlmorpha* Erwin & Geraci, 2008 + *Samiriamorpha* Erwin & Geraci, 2008 (see Erwin and Geraci 2008 for phylogeny).

#### Taxonomy.

Stable at the generic level (Erwin 2013; Erwin and Geraci 2008), although many undescribed species need to be treated (Erwin in prep.). All of these undescribed species have been assigned to the species groups listed herein and their male genitalia have been illustrated, their label data entered into a database, and their localities mapped using Google Earth Pro.

#### Diagnosis.

Form moderately depressed or rarely subcylindrical, narrow or broad, lean or robust, head visible from above, legs concealed beneath when in repose. Color ranges from black to light brown, rarely slightly rufous; only adults of *Pseudomorpha excrucians* Kirby, *Yasunimorpha piranhna* Erwin & Geraci, *Guyanemorpha spectablis* Erwin from Guyane are markedly bicolored. Head with mouthparts visible in dorsal aspect; ventrally beneath eye with deeply recessed groove for insertion of antennal base; mandibular scrobe nearly effaced, delimited by row of short stout setae; mentum and submentum fused; antennal scape partially visible in dorsal aspect. Anterior coxal cavities closed, median coxal cavities conjunct, metepimeron visible. Abdomen with six visible sterna, sternum III with broad medial emargination on posterior margin; sterna V and VI in male with dense row of decumbent and yellowish robust setae medially. Male parameres long, nearly of same length (more or less symmetrical), glabrous or setose, not balteate; phallobase bonnet-shaped, crested or not.

#### Way of life.

As far as is known, adults are found in and around ant nests and in the surrounding vicinity; females are ovoviviparous (Liebherr and Kavanaugh 1985); larvae are known to be ant nest inquilines (Lenko 1972; Erwin 1981), or perhaps living with termites (Ogueta 1967). Of adults found at lights (UV, MV, and white light), most are males.

#### Geographic distribution.

Members of this genus are known to occur from Oregon, Idaho, and Colorado in the north to Argentina in the south, including the Caribbean area, and in southern Australia. They should be looked for in southern Wyoming, where they are also likely to occur. The only eastern species, *Pseudomorpha excrucians* Kirby, is related to species from the Caribbean and South America, not to those lineages from the American west and southwest.

#### Habitat.

Dry loamy or sandy soil where ants prefer to build nests from MASL -72m to 2606m altitude in deserts, grasslands, and open and closed forests.

#### Description.

**Head** (cf. Figs 7–12) with two supraorbital setigerous punctures per eye near their posterior corner, however, numerous accessory setae in some groups obscure them; frontal impressions absent. Clypeus markedly wide, trapezoidal with rounded anterior angles and shallowly lobed posterior margin; posterior margin in some adults very shallow, or effaced, bearing a single long seta each side near anterior corner. Eyes flat, or slightly convex; small gena with numerous stout setae. Antenna of varying length, either shorter, or longer than distance from antennal base to anterior coxae; antennomeres 3–9 slightly wider distally and appearing flattened. Labrum visible, about 2/3 as wide as clypeus, rectangulate, bearing six setae along anterior margin. Mandible markedly flattened with a very short and acute apex; outer margin ventral of the scrobe with short stout setae. Maxillary palpi markedly short, 3-segmented, palpomeres slightly depressed, palpomere 3 truncate apically. Labial palpus with short bisetose palpomere 2; palpomere 3 markedly securiform and robust, its distal margin mostly membranous with sensory organs.

**Figure 1–6.**
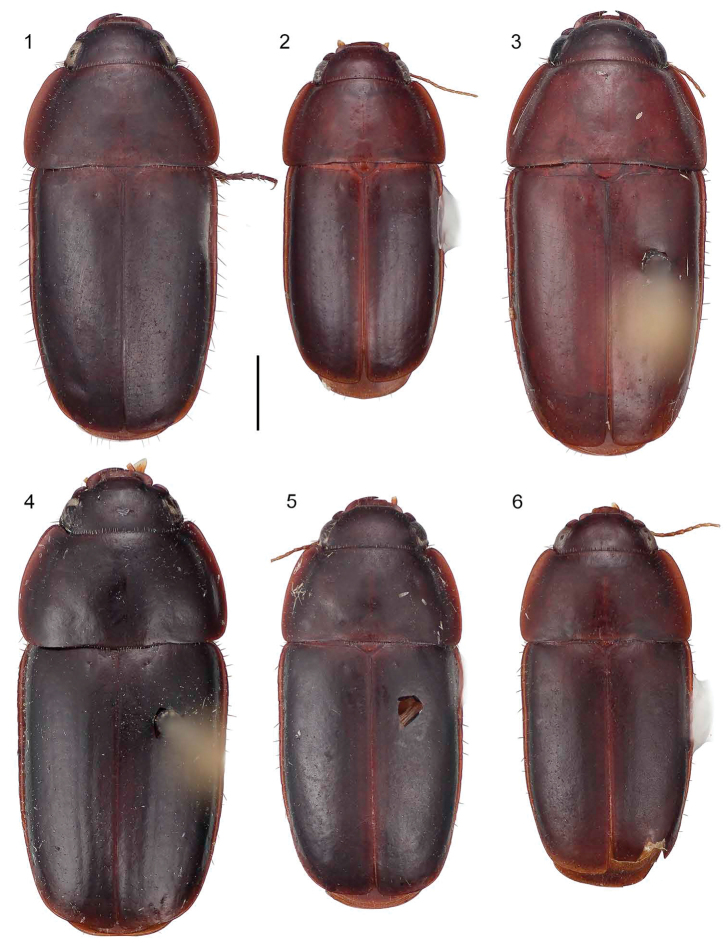
**1** *Pseudomorpha huachinera* sp. n., male holotype, ADP110112; Arroyo El Cocono, Sierra Huachinera, Sonora, México. Habitus, dorsal aspect, ABL = 5.88mm **2** *Pseudomorpha patagonia* sp. n., male holotype, ADP110694; Madera Canyon, Santa Rita Mountains, AZ. Habitus, dorsal aspect, ABL = 4.39mm **3** *Pseudomorpha penablanca* sp. n., female holotype, CAS8111005; Peña Blanca Lake, Arizona. Habitus, dorsal aspect, ABL = 6.37mm **4** *Pseudomorpha pima* sp. n., female holotype, CAS8111006; Madera Canyon (lower), Santa Rita Mountains, Arizona. Habitus, dorsal aspect, ABL = 6.76mm **5** *Pseudomorpha santacruz* sp. n., male holotype, ADP111870; Madera Canyon, Santa Rita Mountains, Arizona. Habitus, dorsal aspect, ABL = 5.78mm **6** *Pseudomorpha santarita* sp. n., male holotype, ADP110817; Pajarito Mountains, AZ. Habitus, dorsal aspect, ABL = 5.33mm.

**Prothorax.** Pronotum (cf. Figs 1–6) wider than head, transverse, with broadly explanate margins, or in cylindrical species narrowly explanate margins; without a pair of setigerous punctures each side, apical, lateral and posterior margins with border of stout setae; hind angles obtuse, broadly rounded. Proepisternum with prosternal process multisetiferous apically, intercoxal process feebly margined.

**Figure 7–12.**
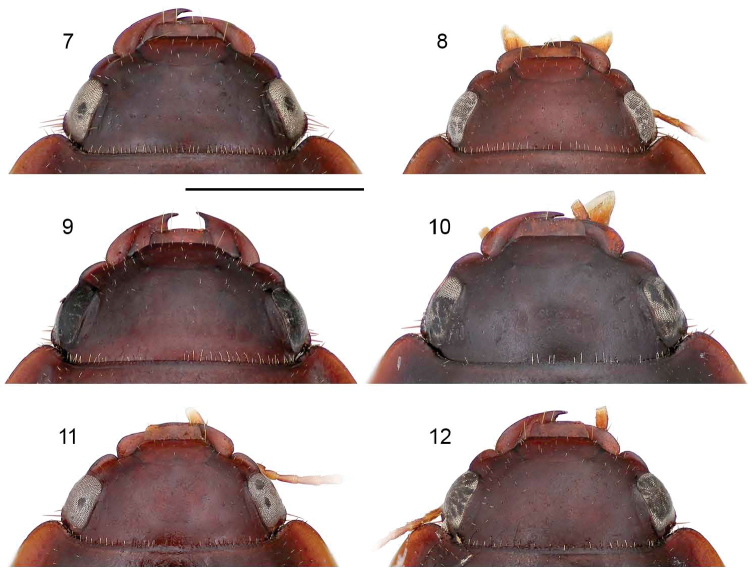
**7** *Pseudomorpha huachinera* sp. n., male holotype, ADP110112; Arroyo El Cocono, Sierra Huachinera, Sonora, México. Head, dorsal planar aspect **8** *Pseudomorpha patagonia* sp. n., male paratype, ADP110694; Madera Canyon, Santa Rita Mountains, AZ. Head, dorsal planar aspect **9** *Pseudomorpha penablanca* sp. n., female holotype, CAS8111005; Peña Blanca Lake, Arizona. Head, dorsal planar aspect **10** *Pseudomorpha pima* sp. n., female holotype, CAS8111006; Madera Canyon (lower), Santa Rita Mountains, Arizona. Head, dorsal planar aspect **11** *Pseudomorpha santacruz* sp. n., male holotype, ADP111870; Madera Canyon, Santa Rita Mountains, Arizona. Head, dorsal planar aspect **12** *Pseudomorpha santarita* sp. n., male holotype, ADP110817; Pajarito Mountains, AZ. A Head, dorsal planar aspect.

**Pterothorax.** Metepisternum elongate though not exceptionally so, the outer margin about 1.5 times greater in length than the anterior margin, posterior margin about 0.5 times anterior margin.

**Elytra.** Elytron rectangulate, slightly narrower apically, wider or narrower (depending on species group) than pronotum at widest point, apical margin subtruncate, outer margin broadly rounded, interneurs present or effaced, of fine or course punctures; parascutellar stria present or absent, parascutellar puncture present, marked; intervals flat to slightly convex without fixed setae, rather variously setiferous, or glabrous. Lateral marginal (umbilical) series of 10–15 setae, concentrated and narrowly spaced in anterior third, widely spaced in posterior two-thirds; lateral margin with border of stout setae.

**Hind wings.** Macropterous. Venation not studied (see Baehr 1997 for illustration of related species, and Erwin 2013).

**Legs.** Short and depressed, femur posteriorly channeled for reception of tibia in repose; antennal comb notch very shallow; tibial spurs normal; anterior tarsi of male with tarsomeres 1–2 dilated slightly, ventrally with two rows of adhesive articulo-setae.

**Abdomen.** Abdominal sterna III-VII with patches of short setae and each of IV–VII with a single row of erect ambulatory setae numbering 2 to 8 setae; V and VI in male with dense row of yellowish robust setae medially.

**Figure 13–17.**
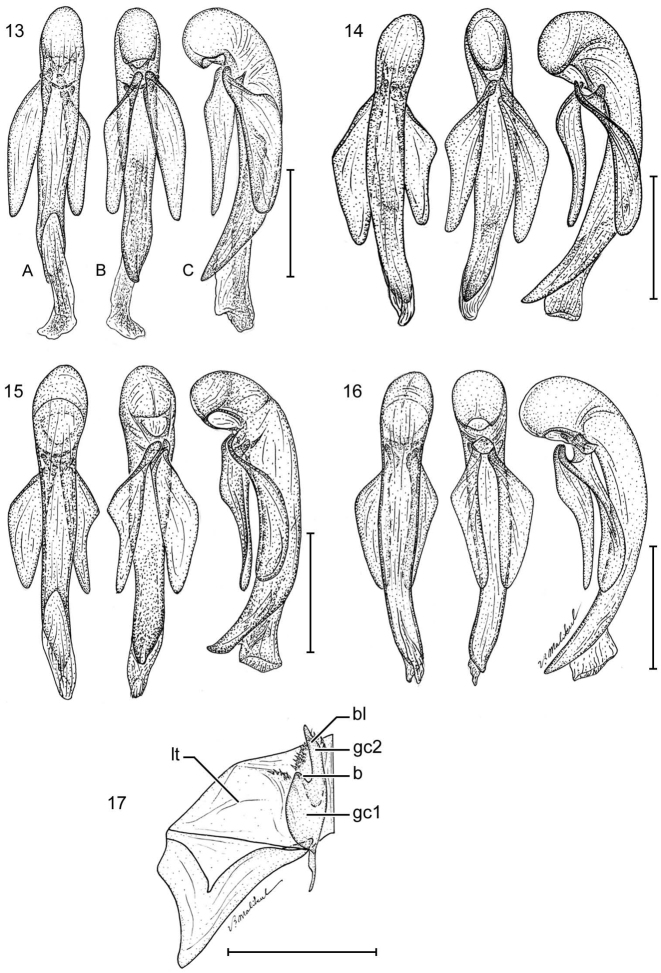
**13** *Pseudomorpha huachinera* sp. n., male paratype, ADP110961; Pajarito Mountains, AZ. Male genitalia, median lobe and parameres labeled as in repose in male, **A** right lateral aspect, **B** left lateral aspect, and **C** dorsal aspects. **14** *Pseudomorpha patagonia* sp. n., male paratype, ADP110694; Harshaw Creek, Patagonia Mountains, AZ **15** *Pseudomorpha santacruz* sp. n., male holotype, ADP111870; Madera Canyon, Santa Rita Mountains, AZ **16** *Pseudomorpha santarita* sp. n., male paratype, ADP110388; Santa Rita Ranch, Santa Rita Mountains, AZ **17** *Pseudomorpha pima* sp. n., female paratype, ADP109219; Bog Springs, Santa Rita Mountains, AZ. Ovipositor sclerites, Legend: **gc1** gonocoxite 1; **gc2** gonocoxite 2; **lt** laterotergite; **b** base of gonocoxite 2; **bl** blade of gonocoxite 2. Scale lines = 0.5 mm.

**Male genitalia** (cf. Figs 13–16). Phallobase hooded with small orifice, dorsum crested or not; phalloshaft straight or markedly arched at basal third, diameter sub-rounded or somewhat depressed dorso-ventrally; phalloapex produced, acute or rounded, depressed dorso-ventrally; endophallus with scattered microtrichia, not in patches. Parameres large, nearly equal in length, left slightly longer and broader than right, each apically glabrous or setiferous.

**Female ovipositor** (cf. Fig. 17). Gonocoxite 2 (**gc 2**) falcate, base (**b**) about as long as blade (**bl**), latter relatively short, pointed distally; margins with several ensiform setae (**en**); with or without short preapical nematiform setae (**n**).

The species groups of *Pseudomorpha* Kirby 1825 and their known distributions (note that some species group names are based on yet undescribed species in Erwin in prep.)

Alleni group. AZ, UT

Augustata group. AZ, CA, NV, NM, TX, UT, México

Behrensi group. CA, CO, ID, NV, NM, OR, UT

Caterinoi group. CA

Consanguinea group. AZ, CA

Cronkhitei group. AZ, CA

Chumash group. CA

Cylindrica group. NM, TX, México

Excrucians group. AR, GA, LA, MS, SC, Argentina, Brazil, Dominican Republic

Falli group. CA

Hubbardi group. AZ, NM, TX

Parallela group. CA, Haiti

Peninsularis group. AZ, CA, CO, NV, NM, OR, UT, México

Phiara group. TX

Pilatei group. TX, Costa Rica, Guatemala, México

Santarita group. AZ, NM, México

Subsulcata group. NM

Tenebroides group. AZ, CA, NV, NM, UT

Vindicata group. CO, ID, UT

##### Key to the species of the *santarita* group of *Pseudomorpha* Kirby 1825

(Taxa referred to in the key are arranged alphabetically in the Species Group Account below)

**Table:** 

1	Pronotum planar, aspect apparently flat with anterior margin on same plane as posterior margin (Figs 1, 2, 4, 5, 6)	2
1’	Pronotum not planar, aspect humped, anterior margin lower (in lateral view) than posterior margin (Fig. 3)	*Pseudomorpha penablanca* Amundson & Erwin, sp. n.
2(1)	Elytron with intervals slightly convex and easily observed with low power magnification (Figs 2, 4)	3
2’	Elytron with intervals effaced and not obvious with low power magnification (Figs 1, 5, 6)	4
3(2)	Small-sized for group, ABL = 4.9 to 5.2 mm, and dark rufous with forebody paler than elytra; margins of elytra parallel in basal two-thirds (Fig. 2)	*Pseudomorpha patagonia* Erwin & Amundson, sp. n.
3’	Large-sized for group, ABL = 6.3 to 6.8 mm, and completely piceous; margins of elytra tapered toward apex (Fig. 4)	*Pseudomorpha pima* Amundson & Erwin, sp. n.
4(2’)	Eye flat in dorsal view, not protruding beyond gena/preocular lobe plane (Figs 7, 11)	5
4’	Eye slightly convex in dorsal view, protruding beyond gena/preocular lobe plane (Fig. 12)	*Pseudomorpha santarita* Erwin & Amundson, sp. n.
5(4)	Pronotum wider than elytra across humeri (Fig. 1); preocular lobe of even width throughout (Fig. 7)	*Pseudomorpha huachinera* Amundson & Erwin, sp. n.
5’	Pronotum slightly narrower than elytra across humeri (Fig. 5); preocular lobe slightly wider anteriorly (Fig. 11)	*Pseudomorpha santacruz* Erwin & Amundson, sp. n.

### Santarita group

**Diagnosis.** With the attributes of the genus as described above and easily recognized by the absence of obvious dorsal setae on head, disc of pronotum, and elytra. Form broad and short, elytra markedly or subtly narrowed toward apex; pronotum coequal or broader than elytra across humeri. Vertex of head without transverse in-line row of coarse setigerous pores, or a band of small setigerous punctulae between eyes, entire surface smooth with sparse minutely setigerous punctulae (cf. Figs 7–12). Eye setiferous or not. Clypeal suture distinct. Pronotum sparsely microsetiferous, without stout setae along lateral margin. Elytron with scutellar setae slightly foveate, interneurs minutely punctulate, punctulae mostly connected by fine longitudinal zigzag striae (high magnification); sterna V and VI of male with broad rows of dense in-line setae, width of rows subequal to length of posterior basitarsomere + 2nd tarsomere; antenna long, extended beyond apex of prosternal process by about the length of last antennomere. Male phallobase without sagittal crest; parameres without setae.

**Status.** At present this group contains six new species described herein.

#### The species of the *santarita* group and their known general distributions.

*Pseudomorpha huachinera* Amundson & Erwin,sp. n., Arizona, México (Sonora)

*Pseudomorpha patagonia* Erwin & Amundson, sp. n., Arizona

*Pseudomorpha penablanca* Amundson & Erwin, sp. n., Arizona

*Pseudomorpha pima* Amundson & Erwin, sp. n., Arizona

*Pseudomorpha santacruz* Erwin & Amundson, sp. n., Arizona

*Pseudomorpha santarita* Erwin & Amundson, sp. n., Arizona, New Mexico.

#### Pseudomorpha (Pseudomorpha) huachinera

Amundson & Erwin sp. n.

http://zoobank.org/DD519173-5E06-420C-92E9-51AC6EF8CE36

http://species-id.net/wiki/Pseudomorpha_huachinera

**Figure 18.**
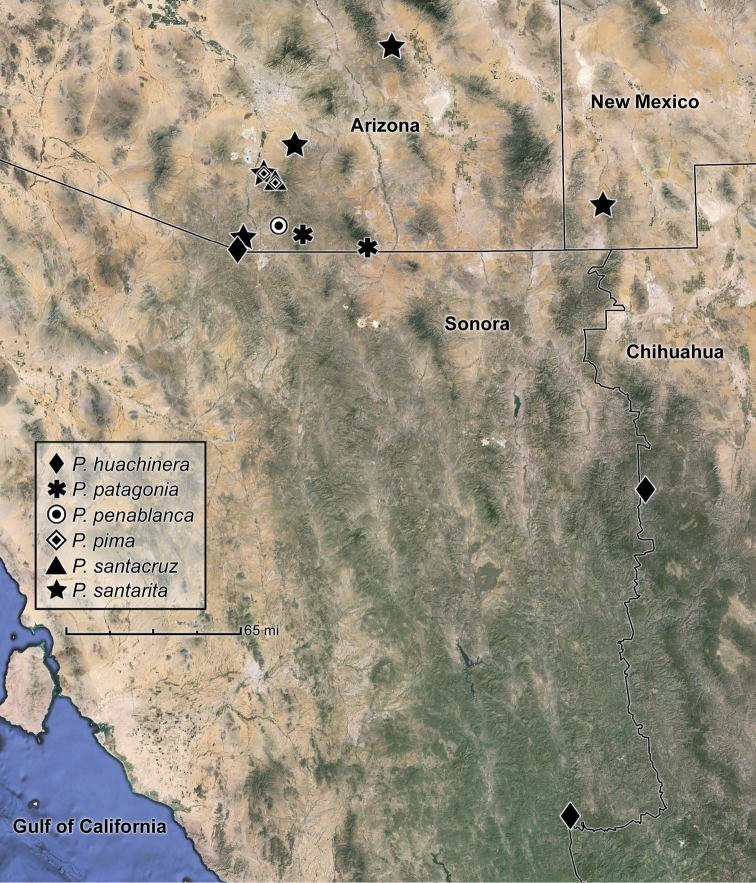
Distribution map for species of the *santarita* group of Pseudomorpha.

Figures 1 , 7 , 13 , 18

##### Holotype.

**México**. Sonora, Nacori Chico, 86.2 km NE Arroyo El Cocono, Sierra Huachinera, 30.044°N, 108.537°W, 1660m, 7–8 August 1982 (G.E. Ball) (NMNH: ADP110112, female). Paratypes are listed below; see other specimens examined.

##### Derivation of scientific epithet.

The epithet “huachinera” is a singular feminine noun used in apposition and refers to the name of the mountain range in which these beetles were collected. The area was once the home of the Ópata Amerindians until the Spanish missionary Cristóbal García founded the town of Juan Evangelista de Huachinera in 1645.

##### Proposed english vernacular name.

Huachinera False-form beetle.

##### Diagnosis.

Color tone of dorsum castaneous and uniform; body rectangulate, lateral margins of elytra parallel, slightly tapering to an apically truncated and laterally slightly rounded apex; dorsum mostly glabrous with irregularly and wide-spaced short erect setae; pronotum with lateral margins broadly explanate, disc convex and medially planar; elytral interneurs and intervals nearly effaced, faint zig-zag interneurs apparent under high magnification, 10 umbilicate setae present near lateral margin, dorsal edge of epipleuron lined with long laterally erect setae.

##### Description.

(Figs 1, 7, 13). Table 1. Size: Medium to large for genus, ABL = 5.9 to 6.4 mm, SBL = 5.9 to 6.4 mm, TW = 4.8 to 5.0 mm. Preocular lobe-eye ratio 0.63 to 0.69. Pronotum ratio (L/W): 0.27 to 0.31. Elytron ratio (L/W): 1.6 to 1.7. *Color*: Dorsum castaneous, explanate margins of pronotum and elytra slightly translucent. *Luster*: Dorsum dull, slightly matte. *Microsculpture*: Small isodiametric sculpticells throughout dorsal surface. **Head:** Genal lobe obsolete, rim posteriad and below eye bearing at least five robust setae directed perpendicular to head; preocular lobe distinct and slightly arching (Fig. 7); eye barely exceeding preocular lobe/gena boundary, barely arcuate; clypeus fused to frons with pigmented furrow slightly effaced medially, bisetose, setae laterad on margin; labrum with four setae projecting anteriorly (Fig. 7); antennal flagellum markedly setose, antennomeres 1-3 bisetose. **Prothorax:** Pronotum mostly glabrous with irregularly and wide-spaced short erect setae, apex slightly rounded medially and narrower than ocular boundary, disk markedly convex and medially planar, width coequal to or slightly wider than elytra across humeri, base and apex fringed with more or less evenly spaced setae, pigmented median line ending about ¾ before basal margin, lateral margins of pronotum with wide explanate sides, anterior angle 72.06°; prosternal apex fringed with short, evenly spaced setae. **Pterothorax:** Scutellum visible, small, triangulate with slightly rounded lateral margins; elytra smooth, interneurs nearly effaced, markedly zig-zagged under high magnification, lateral margin slightly sinuate at basal third, 10 umbilicate erect setae on the ventrally directed curvature of the elytral lateral portion (Fig. 1). **Abdomen:** All sterna sparsely setiferous, sternum III densely so; male sternum IV with broad median dense row of posteriorly decumbent setae, sternum VII with two pair of two setae each along posterior edge; female with 2 pairs of 4 setae on sternum, and numerous longer setae on sterna IV, V, and VI. **Legs:** Legs flattened, setiferous, tibia bearing fringed ring of setae on distal end, femur with distinct lateral sulcus, femora and tibia sparsely setose. **Male Genitalia:** (Fig. 13) Basal orifice hooded by phallobase, orifice recessed and small, phalloshaft arching, shaft narrows toward apex and slightly constricted at apical third; parameres co-equal in length, with the left paramere only slightly longer than right, both asetose; apical orifice small, about 1/5 the length of shaft. **Female Genitalia:** Not investigated.

##### Dispersal potential.

These beetles are macropterous and have been recorded at lights, they are capable of flight; they are swift and agile runners. Accordingly, the species may be expected to be more broadly distributed across a wider geographical range than current records indicate.

##### Way of life.

Adults are likely found in ant nests and the surrounding vicinity; females are ovoviviparous (Liebherr and Kavanaugh 1985); larvae are ant nest inquilines (Erwin 1981). Members of *Pseudomorpha huachinera* occur at midland and upland altitudes in between the Sonoran and Chihuahuan Deserts in the riparian vegetation zones and in oak-pine forests. Adults are active in July– August, very hot months in this area.

##### Other specimens examined.

**México**, Sonora, Yécora, 16 km NW Rancho Aguajia, and 2 km S Old Hwy, 28.403°N, 109.094°W, 1311m, 28–29 July 1987 (S. McCleve) (CAS: 8111009, male paratype). **USA**, Arizona, *Santa Cruz County*, Pajarito Mountains, Pena Blanca Canyon, 31.386°N, 111.093°W, 1191m, 2 July 1980 (S. McCleve) (UATC: ADP110961, male paratype), Pajarito Mountains, Peña Blanca, 31.409°N, 111.085°W, 1283m, 15 August 1964 (R.H. Arnett Jr.) (FSCA: ADP112631, female paratype).

##### Geographic distribution.

(Fig. 18). This species is currently known from Arizona and northern México.

#### Pseudomorpha (Pseudomorpha) patagonia

Erwin & Amundson sp. n.

http://zoobank.org/B32D721B-B86C-4A56-8D4A-CCAAAF8A1318

http://species-id.net/wiki/Pseudomorpha_patagonia

Figures 2 , 8 , 14 , 18

##### Holotype.

**USA**. Arizona, *Santa Cruz County*, Patagonia Mountains, Harshaw Creek, 31.439°N, 110.696°W, 1577m, 1 August 1979 (S. McCleve) (UATC: ADP110694, male).

A paratype is listed below; see other specimens examined.

##### Derivation of scientific epithet.

The epithet “patagonia” is a singular feminine noun used in apposition and refers to the Patagonian Mountain range in Arizona where the type specimen was collected. The area was part of the Apache homeland before being settled by those interested in mining the wealth of minerals nearby.

##### Proposed English vernacular name.

Patagonia False-form beetle.

##### Diagnosis.

Color tone of dorsum alutaceous with head, pronotum and elytral suture paler; body rectangulate, lateral margins of elytra parallel, slightly tapering to an apically truncated and laterally slightly rounded apex; dorsum mostly glabrous with irregularly and wide-spaced short erect setae; pronotum with lateral margins broadly explanate, wider than elytra across humeri, disc markedly convex and medially planar; elytral interneurs minimally impressed yet easily visible under low magnification, 10 umbilicate setae present near lateral margin, dorsal edge of epipleuron lined with long laterally erect setae.

##### Description.

(Figs 2, 8, 14). Table 2. *Size*: Medium for genus, ABL = 4.9 to 5.2 mm, SBL = 4.9 to 5.1 mm, TW = 4.0 to 4.2 mm. Preocular lobe-eye ratio: 0.3 to 0.39. Pronotum ratio (L/W): 0.29. Elytron ratio (L/W): 1.7. *Color*: Dorsum alutaceous with head, pronotum and elytral suture paler. *Luster*: Dorsum dull, slightly matte. *Microsculpture*: Small isodiametric sculpticells throughout dorsal surface.

**Head:** Genal lobe obsolete, rim posteriad and below eye bearing at least five robust setae directed perpendicular to head; preocular lobe distinct and slightly arching (Fig. 8); eye exceeding preocular lobe/gena boundary, shallowly arcuate; clypeus fused to frons with pigmented furrow entire and visible, bisetose, setae laterad on margin; labrum with four setae projecting anteriorly (Fig. 7); antennal flagellum markedly setose, antennomeres 1-3 bisetose.

**Prothorax:** Pronotum (Fig. 2) mostly glabrous with irregularly and wide-spaced short erect setae, apex slightly rounded medially and narrower than ocular boundary, disk markedly convex and medially planar, width coequal to or slightly wider than elytra across humeri, base and apex fringed with more or less evenly spaced setae, pigmented median line ending about ¾ before basal margin, lateral margins of pronotum with wide explanate sides, anterior angle 77.82°; prosternal apex fringed with short, evenly spaced setae. **Pterothorax:** Scutellum visible, small, triangulate with slightly rounded lateral margins; elytra smooth, interneurs very shallow, clearly visible under low magnification, markedly zig-zagged, intervals slightly convex on disc, lateral margin slightly sinuate at basal third or not, 10 umbilicate erect setae on the ventrally directed curvature of the elytral lateral portion (Fig. 2). **Abdomen:** All sterna sparsely setiferous, sternum III densely so; male sternum IV with broad median dense row of posteriorly decumbent setae, sternum VII with two pair of two setae each along posterior edge; female with 2 pairs of 4 setae on sternum, and numerous longer setae on sterna IV, V, and VI. **Legs:** Legs flattened, setiferous, tibia bearing fringed ring of setae on distal end, femur with distinct lateral sulcus, femora and tibiae sparsely setose. **Male Genitalia:** (Fig. 14) Basal orifice hooded by phallobase, orifice recessed and small, phalloshaft arching, shaft narrows toward apex and slightly constricted at apical third; parameres co-equal in length, with the left paramere only slightly longer than right, both asetose; apical orifice small, about 1/5 the length of shaft. **Female Genitalia:** Not investigated.

##### Dispersal potential.

These beetles are macropterous and have been recorded at lights, hence capable of flight; they are swift and agile runners. Accordingly, it is expected that this species be more broadly distributed across a wider geographical range than current records indicate.

##### Way of life.

Adults are likely found in ant nests and the surrounding vicinity; females are ovoviviparous (Liebherr and Kavanaugh 1985); larvae are ant nest inquilines (Erwin 1981). Members of *Pseudomorpha patagonia* occur at upland altitudes in between the Sonoran and Chihuahuan Deserts on oak dominated slopes. See: http://hikearizona.com/photo.php?ZIP=259631. Adults are active in July–August, very hot months in this area.

##### Other specimens examined.

**USA**, Arizona, *Cochise County*, Huachinera Mountains, Copper Canyon, 31.363°N, 110.300°W, 1882m, 16 July 1979 (S. McCleve) (UATC: ADP110736, male paratype).

##### Geographic distribution.

(Fig. 18). This species is currently known from Arizona.

#### Pseudomorpha (Pseudomorpha) penablanca

Amundson & Erwin sp. n.

http://zoobank.org/DE1AD618-8FD9-4138-871F-56ECF6D2B8C9

http://species-id.net/wiki/Pseudomorpha_penablanca

Figures 3 , 9 , 18

##### Holotype.

**USA**: Arizona, *Santa Cruz County*, 3.2 km S of Peña Blanca Lake, 31.473°N, 110.849°W, 1283m, 14–15 August 1971 (W.H. Tyson) (CAS: 8111005, female). Unique.

##### Derivation of specific epithet.

The epithet “penablanca” is a singular feminine noun used in apposition and refers to the lake in Santa Cruz County near the locality at which the holotype was collected. Peña Blanca was built in 1957 by the Arizona Game and Fish Department, and is bordered by oak-savannah hills, some of which are topped with bluffs of limestone.

##### Proposed English vernacular name.

Peña Blanca False-form beetle.

##### Diagnosis.

Color tone of dorsum pale castaneous with head slightly darker; body robust and rectangulate, lateral margins of elytra tapering to an apically truncated and laterally slightly rounded apex; dorsum mostly glabrous with irregularly and wide-spaced short erect setae; pronotum with lateral margins moderately explanate, wider than elytra across humeri, disc markedly convex and medially sloped markedly anteriorly; elytral interneurs minimally impressed yet more or less visible under low magnification, 10 umbilicate setae present near lateral margin, dorsal edge of epipleuron lined with long laterally erect setae.

##### Description.

(Figs 3, 9). Table 3. *Size*: Large for genus, ABL = 6.4 mm, SBL = 6.3 mm, TW = 5.6 mm. Preocular lobe-eye ratio: 0.53. Pronotum ratio (L/W): 0.28. Elytron ratio (L/W): 1.5. *Color*: Dorsum evenly pale rufopiceous except head with notable color gradation from piceous over eyes to rufopiceous medially. *Luster*:Dorsum dull, slightly matte. *Microsculpture*: Small isodiametric sculpticells throughout dorsal surface. **Head:** Genal lobe obsolete, rim posteriad and below eye bearing at least five robust setae directed perpendicular to head; preocular lobe distinct and slightly arching (Fig. 9); eye not exceeding preocular lobe/gena boundary, shallowly arcuate; clypeus fused to frons with pigmented furrow entire and visible, bisetose, setae laterad on margin; labrum with four setae projecting anteriorly (Fig. 9); antennal flagellum markedly setose, antennomeres 1-3 bisetose. **Prothorax:** Pronotum (Fig. 3) mostly glabrous with irregularly and wide-spaced short erect setae, apex straight medially and narrower than ocular boundary, disk markedly convex and medially sloped markedly anteriorly, width slightly wider than elytra across humeri, base and apex fringed with more or less evenly spaced setae, pigmented median line ending about ¾ before basal margin, lateral margins of pronotum with wide explanate sides, anterior angle 71.22°; prosternal apex fringed with short, evenly spaced setae. **Pterothorax:** Scutellum visible, moderate sized, distinctly rounded apically; elytra smooth, interneurs very shallow, clearly visible under medium magnification, markedly zig-zagged, intervals flat on disc, lateral margin very slightly sinuate at basal third, 10 umbilicate erect setae on the ventrally directed curvature of the elytral lateral portion (Fig. 3). **Abdomen:** All sterna sparsely setiferous, sternum III densely so; male unknown; female with 2 pairs of 4 setae on sternum, and numerous longer setae on sterna IV, V, and VI. **Legs:** Legs flattened, setiferous, tibia bearing fringed ring of setae on distal end, femur with distinct lateral sulcus, femora and tibia sparsely setose. **Female Genitalia:** Not investigated.

##### Dispersal potential.

These beetles are macropterous, they are probably capable of flight; they are swift and agile runners. Accordingly, the species is expected to be more broadly distributed across a wider geographical range than current records indicate.

##### Way of life.

Adults are likely found in ant nests and the surrounding vicinity; females are ovoviviparous (Liebherr and Kavanaugh 1985); larvae are ant nest inquilines (Erwin 1981). Members of *Pseudomorpha penablanca* occur at midland altitudes in mountainous areas of Arizona; the holotype was found at night on a dead oak tree. Adults are active in August, a very hot month in this area.

##### Other specimens examined.

None.

##### Geographic distribution.

(Fig. 18). This species is currently known from Arizona.

#### Pseudomorpha (Pseudomorpha) pima

Amundson & Erwin sp. n.

http://zoobank.org/0040741D-5DEE-4BE0-8C86-7C3242251E8D

http://species-id.net/wiki/Pseudomorpha_pima

Figures 4 , 10 , 17 , 18

##### Holotype.

**USA**: Arizona, *Pima County*, Santa Rita Mountains, Lower Madera Canyon, 31.745°N, 110.919°W, 1174m, 14–16 July 1978 (W.H. Tyson) (CAS: 8111006, female). A paratype is listed below; see other specimens examined.

##### Derivation of specific epithet.

The epithet “*pima*” is a singular feminine noun used in apposition and is the name of Aztecan descended peoples that live along the Gila and Salt rivers in southern Arizona.

##### Proposed english vernacular name.

Pima False-form beetle.

##### Diagnosis.

Color tone of dorsum piceous with head slightly darker over eyes; body robust and rectangulate, lateral margins of elytra tapering to an apically truncated and laterally slightly rounded apex; dorsum mostly glabrous with irregularly and wide-spaced short erect setae; pronotum with lateral margins moderately explanate, about coequal in width to that of elytra across humeri, disc markedly convex and planar; elytral interneurs moderately impressed and visible under low magnification, interneurs slightly convex; 10 umbilicate setae present near lateral margin, dorsal edge of epipleuron lined with long laterally erect setae.

##### Description.

(Figs 4, 10, 17). Table 4. *Size*: Large for genus, ABL = 6.3 to 6.8 mm, SBL = 6.3 to 6.7 mm, TW = 5.1 to 5.7 mm. Preocular lobe-eye ratio: 0.56 to 0.57. Pronotum ratio (L/W): 0.31. Elytron ratio (L/W): 1.5 to 1.6. *Color*: Dorsum piceous, slightly lighter brown along explanate edges of dorsum. *Luster*:Dorsum dull, slightly matte. *Microsculpture*: Small isodiametric sculpticells throughout dorsal surface. **Head:** Genal lobe obsolete, rim posteriad and below eye bearing at least five robust setae directed perpendicular to head; preocular lobe distinct, moderately prominent, and more or less straight (Fig. 10); eye not exceeding preocular lobe/gena boundary, shallowly arcuate; clypeus fused to frons with pigmented furrow entire and visible, bisetose, setae laterad on margin; labrum with four setae projecting anteriorly (Fig. 10); antennal flagellum markedly setose, antennomeres 1-3 bisetose. **Prothorax:** Pronotum (Fig. 4) mostly glabrous with irregularly and wide-spaced short erect setae, apex slightly arcuate medially and narrower than ocular boundary, disk markedly convex and planar, width slightly wider than elytra across humeri, base and apex fringed with more or less evenly spaced setae, median line ending about ¾ before basal margin, lateral margins of pronotum with wide explanate sides, anterior angle 89.22°; prosternal apex fringed with short, evenly spaced setae. **Pterothorax:** Scutellum (normally) visible, moderate sized, narrowly rounded apically; elytra smooth (Fig. 4), interneurs very shallow, clearly visible under low magnification, markedly zig-zagged, intervals slightly convex on disc, lateral margin very slightly sinuate at basal third, 10 umbilicate erect setae on the ventrally directed curvature of the elytral lateral portion. **Abdomen:** All sterna sparsely setiferous, sternum III densely so; male unknown; female with 2 pairs of 4 setae on sternum, and numerous longer setae on sterna IV, V, and VI. **Legs:** Legs flattened, setiferous, tibia bearing fringed ring of setae on distal end, femur with distinct lateral sulcus, femora and tibiae sparsely setose. **Female Genitalia:** (see Fig. 17).

##### Dispersal potential.

These beetles are macropterous and have been recorded at lights, hence capable of flight; they are swift and agile runners. Accordingly, the species may be expected to be more broadly distributed across a wider geographical range than current records indicate. Female adults are attracted to lights.

##### Way of life.

Adults are likely found in ant nests and the surrounding vicinity; females are ovoviviparous (Liebherr and Kavanaugh 1985); larvae are ant nest inquilines (Erwin 1981). Members of *Pseudomorpha pima* occur at midland and upland altitudes near the Sonoran Desert. Adults are active in early to mid-July, a very hot month in this area. For images of habitats at the paratype locality (Bog Springs) see: http://www.meetup.com/phoenix-atheists/events/109850282/.

##### Other specimens examined.

**USA**: Arizona, *Pima County*, Santa Rita Mountains, Bog Springs, 31.726°N, 110.874°W, 1524m, 3 July 1958 (J. von Bloeker Jr.) (SBNHM: ADP109219, female paratype).

##### Geographic distribution.

(Fig. 18). This species is currently known from Arizona.

#### Pseudomorpha (Pseudomorpha) santacruz

Erwin & Amundson sp. n.

http://zoobank.org/7D9C4F9B-B6DD-4AEC-A850-6E7F4CFE3C67

http://species-id.net/wiki/Pseudomorpha_santacruz

Figures 5 , 11 , 15 , 18

##### Holotype.

**USA**, Arizona, *Pima County*, Santa Rita Mountains, Madera Canyon, 31.724°N, 110.880°W, 1487m, 11 July 1963 (V.L. Vesterby) (UCDC: ADP111870, male). Unique.

##### Derivation of scientific epithet.

The epithet “santacruz” is a singular masculine noun used in apposition and refers to a river in Arizona where the beetles were found. Before the arrival of Spanish, the area was home to the Apache, Yaqui, and Hohokam peoples who built their communities along what are now called the Santa Cruz River and the Sonoita and Harshaw Creeks.

##### Proposed english vernacular name.

Santa Cruz False-form beetle.

##### Diagnosis.

Color tone of dorsum rufopiceous, lateral margins of pronotum and elytra rufo-translucent; body robust and rectangulate, lateral margins of elytra slightly tapering to an apically truncated and laterally rounded apex; dorsum mostly glabrous with irregularly and wide-spaced short erect setae; pronotum with lateral margins moderately explanate, about coequal in width to that of elytra across humeri, disc markedly convex and planar; elytral interneurs effaced and not visible under medium magnification, intervals flat; 10 umbilicate setae present near lateral margin, dorsal edge of epipleuron lined with long laterally erect setae.

##### Description.

(Figs 5, 11, 15). Table 5. *Size*: Medium for genus, ABL = 5.8 mm, SBL = 5.7 mm, TW = 3.5 mm. Preocular lobe-eye ratio: 0.53. Pronotum ratio (L/W): 0.30. Elytron ratio (L/W): 1.4. *Color*: Dorsum rufopiceous, slightly lighter along explanate edges of pronotum and elytra. *Luster*:Dorsum dull, slightly matte. *Microsculpture*: Small isodiametric sculpticells throughout dorsal surface. **Head:** Genal lobe obsolete, rim posteriad and below eye bearing at least five robust setae directed perpendicular to head; preocular lobe distinct, moderately prominent, and more or less very slightly arcuate (Fig. 11); eye slightly convex, exceeding preocular lobe/gena boundary; clypeus fused to frons with pigmented furrow barely visible, bisetose, setae laterad on margin; labrum with four setae projecting anteriorly (Fig. 11); antennal flagellum markedly setose, antennomeres 1-3 bisetose. **Prothorax:** Pronotum (Fig. 5) mostly glabrous with irregularly and wide-spaced short erect setae laterally, apex very slightly arcuate medially and narrower than ocular boundary, disk markedly convex and planar except for slightly lower apex, width coequal to that of elytra across humeri, base and apex fringed with more or less evenly spaced setae, median line ending about ¾ before basal margin, lateral margins of pronotum with wide explanate sides, anterior angle 77.36°; prosternal apex fringed with short, evenly spaced setae. **Pterothorax:** Scutellum visible, moderate sized, narrowly rounded apically; elytra smooth (Fig. 5), interneurs effaced, not visible under low magnification, intervals flat, lateral margin very slightly sinuate at basal third, 10 umbilicate erect setae on the ventrally directed curvature of the elytral lateral portion. **Abdomen:** All sterna sparsely setiferous, sternum III densely so; male characteristic of species group, see above. Female unknown. **Legs:** Legs all same castaneous color, tibia setiferous with ring of erect yellow setae on distal end. **Male Genitalia:** (Fig. 15) Basal orifice hooded by phallobase, orifice recessed and small, phalloshaft arching, shaft narrows toward apex and slightly constricted at apical third, extreme apex acute and slightly bent; parameres co-equal in length, with the left paramere only slightly longer than right, both asetose; apical orifice small, about 1/5 the length of shaft. **Female Genitalia:** Not investigated.

##### Dispersal potential.

These beetles are macropterous and probably fly; they are swift and agile runners. Accordingly, the species may be expected to be more broadly distributed across a wider geographical range than current records indicate.

##### Way of life.

Adults are likely found in ant nests and the surrounding vicinity; females are ovoviviparous (Liebherr and Kavanaugh 1985); larvae are ant nest inquilines (Erwin 1981). Members of *Pseudomorpha santacruz* occur at midland altitudes near the Sonoran Desert. Adults are active in July, a very hot month in this area.

##### Other specimens examined.

None.

##### Geographic distribution.

(Fig. 18). This species is currently known from Arizona.

#### Pseudomorpha (Pseudomorpha) santarita

Erwin & Amundson sp. n.

http://zoobank.org/2CF69165-EC7A-49E4-8E75-3FC288BAB924

http://species-id.net/wiki/Pseudomorpha_santarita

Figures 6 , 12 , 16 , 18

##### Holotype.

**USA:** Arizona, *Pima County*, Santa Rita Ranch, 31.946°N, 110.758°W, 1080m, July 1978 (R. Lenczy) (NMNH: ADP110388, male). Paratypes are listed below; see other specimens examined.

##### Derivation of specific epithet.

The epithet “*santarita*” is a singular feminine noun used in apposition and is based on the name of the upland range, Santa Rita Mountains in the Coronado National Forest, which includes the type locality of this species. This area was once the home of the indigenous peoples, Papago.

##### Proposed english vernacular name.

Santa Rita False-form beetle.

##### Diagnosis.

With the attributes of the species group as described above and color tawny (Fig. 6), color tone of dorsum uniform; form broad and stout; head with preapical lobe prominent, slightly exceeding gena-eye line; pronotum (Fig. 6) slightly wider at base than elytra across humeri; elytron slightly tapered from humerus to narrower truncated apex and with a bare trace of costae, interneurs of finely impressed zig-zag striae, intervals micropunctate, setae very short, fine, and wide-spaced.

##### Description.

(Figs 6, 12, 16) Table 6. *Size*: Medium for genus, ABL = 5.3 to 5.7 mm, SBL = 5.1 to 5.7 mm, TW = 4.4 to 4.5 mm. Preocular lobe-eye ratio (L/L): 0.551 to 0.693. Pronotum ratio (L/W): 0.225 to 0.286. Elytron ratio (L/W): 1.545 to 1.681. *Color*: Head, pronotum and elytra tawny, their lateral margins somewhat diaphanous rufous, appendages flavotestaceous. *Luster*: Dorsal surface moderately alutaceous, moderately matte. *Microsculpture*: Dorsal surface with very fine flat isodiametric sculpticells. **Head:** (Fig. 12) Eye setiferous. Clypeal suture effaced at middle. Frons and vertex with very sparse micropunctulate, setigerous pores with very short setae widely scattered, no transverse line of setae present. Occiput medial to hind margin of eye without small group of coarse setiferous pores. **Prothorax:** Pronotum (Fig. 6) moderately convex with broad explanate sides, wider than long, without fringe of long stout setae along lateral margin although present at both anterior and hind angles, fringe of short setae present along anterior and posterior margins; anterior margin bead effaced medially, posterior margin discolored but not beaded; disk with longitudinal pigmented line.**Pterothorax:** Elytron (Fig. 6) with interval micropunctate, setigerous pores very widely spaced, finely impressed; interneurs very finely zig-zag striate. Metepisternum longer than wide, surface sparsely setiferous anteriorly. Metasternum sparsely setiferous medially. Metathoracic wing fully developed. **Abdomen:** Sternum III broadly and shallowly incised medially. All sterna sparsely setiferous, IV broadly and densely so medially; male with dense patches of setae medially on sterna V and VI, their width coequal to the combined length of posterior basitarsomere plus tarsomere 2. **Male genitalia:** (Fig. 16) Phallobase crested; phalloshaft arched nearly 90°, then straight and depressed in lateral aspect to phalloapex; phalloapex narrowed both in lateral and dorsal aspects to acute tip. Parameres in ventral aspect with left shorter than right and somewhat narrower, distal margins of both narrowly rounded, asetose.

##### Dispersal potential.

These beetles are macropterous and have been recorded at lights, hence capable of flight; they are swift and agile runners. Accordingly, the species may be expected to be more broadly distributed across a wider geographical range than current records indicate.

##### Way of life.

Adults are likely found in ant nests and the surrounding vicinity; females are ovoviviparous (Liebherr and Kavanaugh 1985); larvae are ant nest inquilines (Erwin 1981). Members of *Pseudomorpha santarita* occur at midland and upland altitudes in between the Sonoran and Chihuahuan Deserts in the riparian vegetation zones with Sycamore (*Platanus occidentalis* L.) and Cottonwood (*Populus Fremontii* S. Wats.) and desert scrub on the slopes. Adults are active in July –August, very hot months in this area.

##### Other specimens examined.

**USA**: Arizona, *Santa Cruz County*, Pajarito Mountains, Peña Blanca Canyon, 31.386°N, 111.093°W, 1191m, 2 July 1980 (S. McCleve) (UATC: ADP110817, male paratype); *Graham County*, Galiuro Mountains north, Ash Creek, 32.514°N, 110.139°W, 1400m, 16–17 August 1982 (D.R. Maddison, G.E. Ball & S. McCleve) (DRMC: ADP110591, male paratype); *Pima County*, Santa Rita Mountains, Madera Canyon, 31.724°N, 110.880°W, 1487m, 11 July 1963 (V.L. Vesterby) (UCDC: ADP111898, female paratype), Madera Canyon, 1499m, 31.724°N, 110.880°W, 8 July 1970 (K. Stephan) (FSCA: ADP112570, male paratype). New Mexico, *Hidalgo County*, Animas Mountains, Double Adobe Creek, 31.614°N, 108.779°W, 1755m, 11 July 1981 (S. McCleve) (UATC: ADP110861, male paratype).

##### Geographic distribution.

(Fig. 18). This species is currently known from Arizona and New Mexico.

## Concluding statement

In studies of one the of top five most speciose families of beetles, the Carabidae, with nearly 40,000 described species (Lorenz 2005), focusing on beetles that live with ants provides several additional dimensions of field work and commensal investigations beyond that of studying free roaming predators (most carabids) and the seed-eaters (Harpalini and Zabrini). The present revision, in tandem with Erwin and Geraci (2008), provides a starting point in getting many names and descriptions for several new species described so they can be included in an upcoming synopsis of the genus for the Western Hemisphere (Erwin in prep.). Although the genus will be rather straight-forward, learning more about the ways of life of included species will be far more difficult. Future students of “beetles that live with ants” will be digging up ant nests to determine host specificity of the beetle species and to begin the task of understanding the way of life of the immature stages. Given that far more males than females have been collected and they are overwhelmingly the ones coming to the UV, MV, and white lights of collectors, one must wonder how much fidelity females have to their host ant’s nest. Do females really disperse much at all? What chemical magic do larvae have that keep them safe and fed in the brood chambers of ants? What role do the unique cephalic setae (Erwin 1981) of larvae play inside the nest? With the ability to “tuck everything in” (i.e., legs and antennal attributes of adult pseudomorphines), it seems they are well adapted to living with aggressive ants. Does this mean that males also frequent ant nests? Is that where mating occurs? The pseudomorphines are a very interesting evolutionary off-shoot of the typical carabid morphotype in both form and function and are only just now beginning to be understood in North America. The fact that species of related genera in South America are living with arboreal ants will make learning about those species even more difficult. Insecticidal fogging of the canopy produces adults of these species, but only tearing apart arboreal *Azteca* ant nests, while suspended in a tree, will produce their larvae; and that is not for carabidologists faint of heart.

## Supplementary Material

XML Treatment for Pseudomorphini

XML Treatment for Pseudomorpha

XML Treatment for Pseudomorpha (Pseudomorpha) huachinera

XML Treatment for Pseudomorpha (Pseudomorpha) patagonia

XML Treatment for Pseudomorpha (Pseudomorpha) penablanca

XML Treatment for Pseudomorpha (Pseudomorpha) pima

XML Treatment for Pseudomorpha (Pseudomorpha) santacruz

XML Treatment for Pseudomorpha (Pseudomorpha) santarita

## Appendix 1

Institutions and personnel who loaned specimens for this revision.

CAS David H. Kavanaugh, Department of Entomology, California Academy of Science, 55 Music Concourse Drive, San Francisco, CA 94118 U.S.A.

DRMC David R. Maddison, Department of Zoology, 3029 Cordley Hall, Oregon State University, Corvallis, OR 97331 USA

FSCA Paul E. Skelley, Florida State Collection of Arthropods, Division of Plant; Industry/Entomology, Florida Department of Agriculture and Consumer Services, The Doyle; Conner Building, 1911 SW 34th St., Gainesville, FL 32608 USA

SBNHM Zachary H. Falin, Division of Entomology, KU Biodiversity Research Center, 1501; Crestline Dr., Suite #140, University of Kansas, Lawrence, KS 66045-4401, USA

UASM George E. Ball, Danny Shpeley, Strickland Entomological Museum, CW-405 Biological Sciences Building, University of Alberta, Edmonton, Alberta T6G 2E9 CANADA

UATC Wendy Moore, Department of Entomology, University of Arizona, 1140 E. South; Campus Dr., Forbes 410, Tucson, AZ 85721-0036 USA

UCDC Lynn Kimsey, Bohart Museum of Entomology, University of California, One Shield Ave., Davis, CA 95616 USA

## Appendix 2

**Table 1.** Tables with measurements and ratios for *Pseudomorpha huachinera* sp. n.; all measurements are in millimeters.

**Total length**					
Males			Females		
N	Range	Mean	N	Range	Mean
2	6.29–6.414	6.352	2	5.866–6.022	5.944
**Maximum width**					
Males			Females		
N	Range	Mean	N	Range	Mean
2	4.798–4.82	4.809	2	4.784–5.018	4.901
**W of head/w of l. Elytron**					
Males			Females		
N	Range	Mean	N	Range	Mean
2	1.257–1.258	1.257	2	1.185–1.227	1.206
**Pronotum: width (at widest part)/length**					
Males			Females		
N	Range	Mean	N	Range	Mean
2	3.177–3.466	3.321	2	3.294–3.694	3.496
**Length of pronotum / length of head**					
Males			Females		
N	Range	Mean	N	Range	Mean
2	2.068–2.327	2.197	2	2.170–2.977	2.574

**Table 2.** Tables with measurements and ratios for *Pseudomorpha patagonia* sp. n.; all measurements are in millimeters.

**Total length**					
Males			Females		
N	Range	Mean	N	Range	Mean
2	4.899–4.964	4.932	0		
**Maximum width**					
Males			Females		
N	Range	Mean	N	Range	Mean
2	3.98–4.206	4.093	0		
**W of head/w of l. Elytron**					
Males			Females		
N	Range	Mean	N	Range	Mean
2	1.196–1.249	1.223	0		
**Pronotum: width (at widest part)/length**					
Males			Females		
N	Range	Mean	N	Range	Mean
2	3.450–3.523	3.487	0		
**Length of pronotum / length of head**					
Males			Females		
N	Range	Mean	N	Range	Mean
2	2.261–2.753	2.507	0		

**Table 3.** Tables with measurements and ratios for *Pseudomorpha penablanca* sp. n.; all measurements are in millimeters.

**Total length**					
Males			Females		
N	Range	Mean	N	Range	Mean
0			1	6.37	
**Maximum width**					
Males			Females		
N	Range	Mean	N	Range	Mean
0			1	5.642	
**W of head/w of l. Elytron**					
Males			Females		
N	Range	Mean	N	Range	Mean
0			1	1.119	
**Pronotum: width (at widest part)/length**					
Males			Females		
N	Range	Mean	N	Range	Mean
0			1	3.414	
**Length of pronotum / length of head**					
Males			Females		
N	Range	Mean	N	Range	Mean
0			1	2.566	

**Table 4.** Tables with measurements and ratios for *Pseudomorpha pima* sp. n.; all measurements are in millimeters.

**Total length**					
Males			Females		
N	Range	Mean	N	Range	Mean
0			2	6.338–6.72	6.529
**Maximum width**					
Males			Females		
N	Range	Mean	N	Range	Mean
0			2	5.124–5.66	5.392
**W of head/w of l. Elytron**					
Males			Females		
N	Range	Mean	N	Range	Mean
0			2	1.148–1.195	1.172
**Pronotum: width (at widest part)/length**					
Males			Females		
N	Range	Mean	N	Range	Mean
0			2	3.164–3.284	3.224
**Length of pronotum / length of head**					
Males			Females		
N	Range	Mean	N	Range	Mean
0			2	2.221–2.358	2.289

**Table 5.** Tables with measurements and ratios for *Pseudomorpha santacruz* sp. n.; all measurements are in millimeters.

**Total length**					
Males			Females		
N	Range	Mean	N	Range	Mean
1	5.7866		0		
**Maximum width**					
Males			Females		
N	Range	Mean	N	Range	Mean
1	5.138		0		
**W of head/w of l. Elytron**					
Males			Females		
N	Range	Mean	N	Range	Mean
1	1.122		0		
**Pronotum: width (at widest part)/length**					
Males			Females		
N	Range	Mean	N	Range	Mean
1	3.432		0		
**Length of pronotum / length of head**					
Males			Females		
N	Range	Mean	N	Range	Mean
1	2.441		0		

**Table 6.** Tables with measurements and ratios for *Pseudomorpha santarita* sp. n.; all measurements are in millimeters.

**Total length**					
Males			Females		
N	Range	Mean	N	Range	Mean
4	5.133-5.733	5.443	1	5.636	
**Maximum width**					
Males			Females		
N	Range	Mean	N	Range	Mean
4	3.958-4.428	4.202	1	4.466	
**W of head/w of l. Elytron**					
Males			Females		
N	Range	Mean	N	Range	Mean
4	1.229-1.365	1.282	1	1.258	
**Pronotum: width (at widest part)/length**					
Males			Females		
N	Range	Mean	N	Range	Mean
4	3.071-3.545	3.334	1	3.471	
**Length of pronotum / length of head**					
Males			Females		
N	Range	Mean	N	Range	Mean
4	1.800-2.471	2.161	1	2.675	
